# Bevacizumab in First-Line Chemotherapy Improves Progression-Free Survival for Advanced Ovarian Clear Cell Carcinoma

**DOI:** 10.3390/cancers13133177

**Published:** 2021-06-25

**Authors:** Shinichi Tate, Kyoko Nishikimi, Ayumu Matsuoka, Satoyo Otsuka, Yuki Shiko, Yoshihito Ozawa, Yohei Kawasaki, Makio Shozu

**Affiliations:** 1 Department of Gynecology, Chiba University Hospital, 1-8-1 Inohana, Chuo-ku, Chiba 260-8677, Japan; knishikimi@hospital.chiba-u.jp (K.N.); a-matsuoka@chiba-u.jp (A.M.); caxa5597@chiba-u.jp (S.O.); shozu@faculty.chiba-u.jp (M.S.); 2 Biostatistics Section, Clinical Research Center, Chiba University Hospital, 1-8-1 Inohana, Chuo-ku, Chiba 260-8677, Japan; shiko_yuki@chiba-u.jp (Y.S.); yoshihito.ozawa@chiba-u.jp (Y.O.); ykawasaki@chiba-u.jp (Y.K.)

**Keywords:** bevacizumab, chemotherapy, ovarian cancer, progression-free survival, surgery

## Abstract

**Simple Summary:**

We investigated survival outcomes following first-line chemotherapy before and after approval of bevacizumab for ovarian cancer in Japan to evaluate the efficacy of bevacizumab for advanced clear cell carcinoma. We investigated 28 consecutive patients diagnosed with clear cell carcinoma (stages III/IV) at our hospital between 2008 and 2018. Bevacizumab was administered for treatment after approval in Japan in November 2013. Progression-free survival was compared between 10 patients treated before bevacizumab approval (2008–2013,) and 18 patients treated after Bev approval (2014–2018) for first-line chemotherapy. The median progression-free survival increased from 12.0 months before bevacizumab approval to 29.8 months after bevacizumab approval (Wilcoxon test, *p* = 0.026). Multivariate analysis showed that performance status (*p* = 0.049), bevacizumab administration (*p* = 0.023) and completeness of resection (*p* = 0.023) were independent prognostic factors for progression-free survival. Bevacizumab incorporated into first-line chemotherapy might improve progression-free survival in patients with advanced clear cell carcinoma.

**Abstract:**

(1) Background: We investigated survival outcomes following first-line chemotherapy before and after approval of bevacizumab (Bev) for ovarian cancer in Japan to evaluate the efficacy of Bev for advanced clear cell carcinoma (CCC). (2) Methods: We investigated 28 consecutive patients diagnosed with CCC (stages III/IV) at our hospital between 2008 and 2018. Bev was administered for treatment of advanced CCC after approval in Japan in November 2013. Progression-free survival (PFS) was compared between 10 patients treated before Bev approval (2008–2013, Bev- group) and 18 patients treated after Bev approval (2014–2018, Bev+ group) for first-line chemotherapy. (3) Results: No intergroup difference was observed in patient characteristics. The rate of completeness of resection was higher in the Bev − group (9/10, 90%) than in the Bev+ group (15/18, 83%) (*p* = 0.044). Eleven (61%) patients in the Bev + group received ≥ 21 cycles of Bev. The median PFS increased from 12.0 months before Bev approval to 29.8 months after Bev approval (Wilcoxon test, *p* = 0.026). Multivariate analysis showed that performance status (*p* = 0.049), Bev administration (*p* = 0.023) and completeness of resection (*p* = 0.023) were independent prognostic factors for PFS. (4) Conclusions: Bev incorporated into first-line chemotherapy might improve PFS in patients with advanced CCC. We hope that our findings will be confirmed in adequate clinical trials.

## 1. Introduction

Clear cell carcinoma (CCC), a subtype of epithelial ovarian carcinoma, is rare in Europe and the United States [1]; however, it accounts for 20–30% of epithelial ovarian tumors in Asia, particularly in Japan [2,3]. Many CCCs are often diagnosed at an early stage, and advanced disease is uncommon. The Japanese Gynecologic Oncology Group (JGOG) 3017/Gynecologic Cancer Intergroup (GCIG) trial [4] reported that advanced CCC was diagnosed in only 156/667 patients (23.3%). CCC shows a poorer prognosis than high-grade serous carcinoma [1,3,5], particularly in patients with advanced-stage disease because it is less sensitive to conventional platinum-based chemotherapy [2,5,6,7]. Reportedly, paclitaxel-containing platinum chemotherapy was more effective than conventional platinum chemotherapy for advanced CCC [8]. However, no cytotoxic regimen more effective than paclitaxel and carboplatin has been reported in the available literature [4]. Therefore, therapeutic strategies for advanced CCC are urgently warranted.

The efficacy of bevacizumab (Bev) for CCC remains unknown because of the low incidence of this cancer. Two phase 3 trials have reported that the addition of Bev during and after carboplatin and paclitaxel chemotherapy prolonged progression-free survival (PFS) by approximately four months in patients with advanced epithelial ovarian cancer [9,10]. However, the exploratory subgroup analysis did not show any specific group of patients in whom Bev was particularly effective for treatment [11]. There are various mechanisms for induction of VEGF in CCC. The effectiveness of Bev in patients with CCC is attributable to Bev-induced AKT-mTOR pathway inhibition [12]. It has been reported that mTOR was more frequently activated in CCC than in serous adenocarcinoma [13]. In addition to activation of the mTOR pathway, endometriotic tumor microenvironment is closely associated with hypoxic condition in CCC [14]. Hypoxia-inducible factor (HIF) 1α expression levels are significantly higher in CCC than in other histologic subtypes of ovarian cancer [12]. This pathway is known to promote the expression of HIF 1α, which mediates the activation of vascular endothelial growth factor (VEGF), a potent pro-angiogenic factor necessary for tumor growth, invasion, and metastasis [15]. Although a few studies have reported the efficacy of Bev in patients with recurrent CCC [16,17] and high response rates of CCC to Bev during initial chemotherapy [18], no previous report has described the survival impact of Bev in first-line treatment.

The survival impact of Bev combined with aggressive surgery is also unknown in patients with advanced CCC. Aggressive surgery appears to be a more important treatment for advanced CCC than for serous carcinoma due to less chemo-sensitive [19]. Some retrospective studies have reported that complete resection without residual disease is the only determinant of improved survival outcomes in patients with advanced CCC [20]. Some gynecologic oncologists believe that aggressive surgery may be unnecessary during Bev administration, while others propose that aggressive surgery can obviate the need for Bev administration. Aggressive surgery, including gastrointestinal and upper abdominal procedures, was introduced for advanced ovarian cancer at our hospital in 2008. We observed that aggressive surgery performed mainly by gynecologic oncologists was safe and prolonged survival [21]. Moreover, Bev administration was initiated since it was approved for recurrent and advanced ovarian cancer in Japan in 2014 [22]. In this study, we retrospectively investigated the efficacy of Bev for advanced CCC and compared survival outcomes pre- and post-Bev approval after aggressive surgery was introduced at our hospital.

## 2. Materials and Methods

### 2.1. Patients

This retrospective, case-control study included 28 patients diagnosed with stage III/IV CCC (International Federation of Gynecology and Obstetrics [FIGO 2014] classification [23]), who were treated at Chiba University Hospital between January 2008 and December 2018. We performed cytoreductive surgery followed by first-line chemotherapy. Neoadjuvant chemotherapy was followed by interval debulking surgery in patients in whom primary debulking surgery was contraindicated. Aggressive surgery was introduced at our hospital for the treatment of advanced ovarian cancer in 2008, and gastrointestinal and upper abdominal surgeries were performed by gynecologic oncologists to achieve complete resection. Procedural details with regard to aggressive surgery are described in our previous report [21]. From a prospectively enrolled database of patients who underwent surgery and chemotherapy at our hospital, we obtained patients’ clinical data including age, FIGO stage, performance status (PS), peritoneal cancer index (PCI) [24], timing of debulking surgery, Bev administration, the number of Bev cycles administered, completeness of debulking surgery, the surgical complexity score [25], and adverse events associated with Bev-containing chemotherapy administered along with aggressive surgery. PCI is calculated as a sum of peritoneal tumor sizes in 13 different abdominopelvic regions [24]. The surgical complexity score rates each method used for ovarian cancer surgery, and the sum of this score represents the complexity of each procedure [25]. Bev (15 mg/kg every 3 weeks) was administered after it was approved in Japan in November 2013 for the treatment of FIGO III/IV ovarian cancer. The PFS and overall survival (OS) rates were compared between 10 patients treated before Bev approval (2008–2013, Bev- group) and 18 patients treated after Bev approval (2014–2018, Bev+ group). The study was approved by the Institutional Review Board (#3735) of Chiba University.

### 2.2. Selection Criteria for Primary Debulking Surgery or Neoadjuvant Chemotherapy Followed by Interval Debulking Surgery in Our Institute

When the surgical team (ST, KN, AM) deemed that complete resection was achievable by including upper abdominal surgery, the surgeon performed primary debulking surgery followed by six courses of adjuvant chemotherapy. If the surgeon deemed complete resection as impossible or life-threating, or that neoadjuvant chemotherapy would be preferable in terms of a trade-off between therapeutic efficacy and safety, the surgeon triaged the patient to a neoadjuvant chemotherapy followed by interval debulking surgery subgroup and performed a diagnostic biopsy with or without tumorectomy and/or partial omentectomy for symptom relief (e.g., for massive ascites).

### 2.3. Diagnosis of Recurrence

Computed tomography (CT) or positron emission tomography (PET) was used to diagnose recurrence. Serum cancer antigen 125 (CA125) levels were measured at 1–2-month intervals for the first 2 years and at 3-month intervals between the 3rd and 5th years after treatment completion. CT was performed at the end of initial treatment and every 6 months thereafter. CT was also performed in patients who showed increased serum CA125 levels or worsening of symptoms. Patients were closely followed-up and underwent PET/CT if CT failed to detect recurrence despite increased serum CA125 levels. 

### 2.4. Chemotherapy and Bevacizumab

A combination of taxane-platinum was used as first-line chemotherapy in this study. Triweekly paclitaxel (175 mg/m^2^) plus carboplatin (area under the curve [AUC] 5–6) or triweekly docetaxel (75 mg/m^2^) plus carboplatin (AUC 5–6) was administered in 2008–2009. Weekly paclitaxel (80 mg/m^2^/week injected intravenously) and carboplatin (AUC 2–3/week injected intravenously) were administered between 2010 and 2018. Bev (15 mg/kg every 3 weeks) was administered to patients without any contraindication to its use after this drug was approved in November 2013 in Japan for the treatment of ovarian cancer. The Common Terminology Criteria for Adverse Events scale, version 4.0 published by the National Cancer Institute was used to grade toxicity.

### 2.5. Statistical Analysis 

The PFS and OS were the primary and secondary endpoints, respectively. The Kaplan–Meier method was used to estimate the PFS, OS, and the time until the first recurrence detected at intra- and extraperitoneal sites. The log-rank and Wilcoxon tests were used to compare statistically significant differences. PFS was defined as the time interval between the date of treatment initiation and the date of diagnosis of the first recurrence. OS was defined as the time interval between treatment initiation and the date of death or the last follow-up. Patient characteristics were compared between the Bev- and Bev+ groups using the Fisher exact test or the Chi-square test. The variables included the multivariate analysis were selected using backward stepwise selection based on the corrected Akaike’s information criterion. Performance status, bevacizumab use, and completeness of resection were selected. Using three variables, Cox proportional hazards regression analysis was performed to analyze the prognostic factors associated with PFS and OS. All statistical analyses were two-sided, and a *p* value < 0.05 was considered statistically significant. All statistical analyses were performed using the JMP statistical software, version 11.0 (SAS, Cary, NC, USA).

## 3. Results

### 3.1. Patient Characteristics

Table 1 shows patient characteristics; no statistically significant intergroup difference was observed in patient characteristics. The median age was 54 years and 53 years in the Bev− and Bev+ groups (*p* = 0.810), respectively. Stage IV disease was diagnosed in 3 (30%) and 4 (22%) patients in the Bev− and Bev+ groups, respectively (*p* = 0.674). The PS was ≥ 2 in 1 (10%) and 5 (28%) patients in the Bev– and Bev+ groups, respectively (*p* = 0.375). The peritoneal cancer index was 5.5 in both groups (*p* = 0.727). Primary debulking surgery was performed in 10 (100%) and 11 (61%) patients in the Bev− and Bev+ groups, respectively. The median surgical complexity score was 9 in both groups (*p* = 0.700). The rate of completeness of resection was higher in the Bev− group (9/10, 90%) than in the Bev+ group (15/18, 83%) (*p* = 0.044). One patient in the Bev+ group could not complete first-line chemotherapy because of disease progression.

### 3.2. Bevacizumab Administration

We administered a median of 21 cycles (interquartile range (IQR) 7.5–21) of Bev, and 12 of 18 patients (67%) completed Bev maintenance therapy. Notably, 12 of 18 patients in the Bev+ group showed recurrence, and 6 of these patients underwent Bev re-administration after disease progression. The remaining six patients did not receive Bev because of poor PS or development of adverse events. In the Bev- group, relapse occurred in 7 of 10 patients; 4 of these patients received Bev for relapse.

### 3.3. Survival Analysis

**Figure 1:**
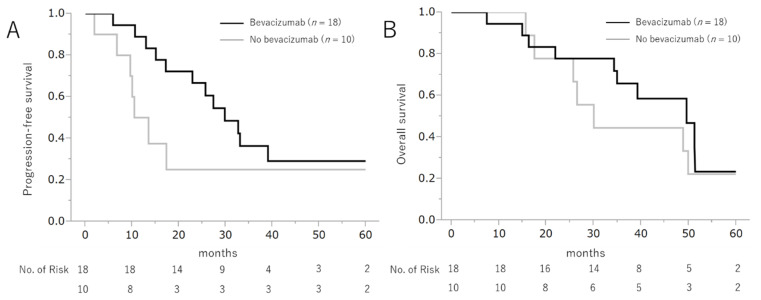
Progression-free survival and overall survival following the addition of bevacizumab to the chemotherapeutic regimen.

Figure 1 shows the PFS and OS rates. The median follow-up period was 36.8 months (IQR 22.4–49.9). The median PFS in the Bev+ and Bev- groups was 29.8 months (95% confidence interval [CI] 17.2–infinity) and 12.0 months (95% CI 1.9–infinity) (log-rank test *p* = 0.156 and Wilcoxon test *p* = 0.036), respectively. The median OS in the Bev+ and Bev− groups was 49.6 months (95% CI 34.3–infinity) and 30.0 months (95% CI 15.6–infinity) (log-rank test *p* = 0.530 and Wilcoxon test *p* = 0.464), respectively.

### 3.4. Bevacizumab-Induced Adverse Events

Table 2 shows the Bev-induced adverse events observed in this study. Adverse events included grade 2 and 3 hypertension (33% and 22%, respectively), grade 2 and 3 proteinuria (6% and 22%, respectively), and grade 3 bleeding (6%). No patient developed thromboembolic events, gastrointestinal perforation, or fistula.

### 3.5. Perioperative Complications

Severe perioperative complications (Clavien-Dindo grade ≥ IIIb [26]) occurred in 2 patients (7.1%, grade IIIb: lymphorrhea and intra-abdominal bleeding in 1 patient each).

### 3.6. Multivariate Analysis of Prognostic Factors Associated with Progression-Free Survival

Cox proportional hazards regression analysis was performed to analyze the prognostic factors associated with PFS and OS (Table 3). Performance status (hazard ratio [HR] 0.28, 95% CI 0.09–0.94, *p* = 0.049), Bev administration (HR 0.26, 95% CI 0.08–0.82, *p* = 0.022) and completeness of resection (HR 0.20, 95% CI 0.06–0.70, *p* = 0.013) were independently associated with the PFS, and completeness of resection (HR 0.19, 95% CI 0.05–0.78, *p* = 0.021) was independently associated with OS.

## 4. Discussion

### 4.1. Key Findings of This Study

In this study, we observed that the addition of Bev to the chemotherapeutic regimen and completeness of resection were associated with successful initial treatment of advanced CCC. Despite its small sample size, this study highlights the effectiveness of an important treatment strategy for the initial treatment of advanced CCC. To date, even large-scale clinical studies have not conclusively established the efficacy of Bev for the treatment of advanced CCC owing to the low incidence of this cancer. In this study, we confirmed that anti-angiogenic therapy that targets the VEGF pathway as well as aggressive surgery could effectively treat advanced CCC. Our results are consistent with exploratory subgroup analyses performed in large clinical trials, which prove that Bev had consistently better effects on any factors [9].

### 4.2. Efficacy of Bev in First-Line Chemotherapy

We observed that the addition of Bev to the chemotherapeutic regimen prolonged the PFS of patients with advanced CCC. Several large randomized controlled trials have reported the efficacy of Bev addition to first-line chemotherapy for advanced ovarian cancer [9,10]. However, these trials enrolled a small number of patients with CCC because these studies included patients from Western countries, and exploratory subgroup analyses could not establish the efficacy of Bev in non-serous ovarian cancer [9]. The ICON-7 trial could prove the efficacy of Bev with regard to the PFS and OS in the high-risk group; however, the role of Bev in patients with CCC, including in those with stage I–IV disease could not be definitively established primarily because >50% of patients with CCC included in this trial were diagnosed with early-stage ovarian cancer (stages I and II) [27]. A prospective observational study that investigated first-line chemotherapy showed that paclitaxel-carboplatin combination chemotherapy with the addition of Bev for CCC was associated with a response rate of 63.6% (*n* = 11), suggesting that the addition of Bev could play a key role in the optimization of treatment for CCC [18]. Our study is the first to highlight that the addition of Bev to the initial chemotherapeutic regimen prolonged PFS in patients with advanced CCC.

### 4.3. Efficacy of Bev in Salvage Chemotherapy

In addition to its role in first-line chemotherapy, a few reports have described the effects of Bev added to salvage chemotherapy regimens for the treatment of CCC. In clinical trials that investigated patients with recurrent ovarian cancer (both platinum-sensitive [28,29] and resistant disease [30]), CCC was diagnosed in ≤10% of the study population. Retrospective studies that investigated the effects of Bev on CCC observed that Bev added to salvage chemotherapy regimens for recurrent CCC was associated with high response rates and prolonged PFS [31]. Similarly, Bev monotherapy was also associated with high response rates and prolonged PFS [16]. A case report in the available literature has described that Bev administered to a patient with CCC and pericardial and pleural effusion improved the patient’s symptoms and quality of life [17]. 

### 4.4. Efficacy of Aggressive Surgery in CCC

Several gynecologic oncologists are of the view that novel molecularly targeted therapy using agents such as Bev could obviate the need for aggressive surgery as initial treatment because secondary debulking surgery did not result in longer PFS and OS than chemotherapy with Bev in GOG-213 trial [32]. However, CCC is less sensitive to chemotherapy; therefore, aggressive surgery may be warranted for the initial treatment of CCC. Aggressive surgery was shown to be effective in advanced CCC [20], similar to its role in other histopathological cancer types [33,34,35]. The ICON-7 [10] trial confirmed a 2-month prolongation in PFS, and the GOG-218 trial [9] confirmed a 3.8-month prolongation in PFS, following Bev administration. We observed a 17-month prolongation in PFS in our study. Although it is difficult to compare the results of the two clinical trials, which primarily enrolled patients with serous carcinoma, the significant prolongation in PFS observed in our study cannot be attributed exclusively to the addition of Bev to the therapeutic regimen of patients with CCC, who showed a >85% complete resection rate. The ICON 7 trial reported that the addition of Bev was effective in patients with advanced ovarian cancer, who underwent complete resection or even in patients with residual disease. Therefore, it is reasonable to conclude that the addition of Bev and aggressive surgery may have synergistic effects on PFS, while the completeness of surgical resection was the only independent factor associated with OS.

### 4.5. Strengths and Limitation

Following are the limitations of the current study: (A) The retrospective design of this small-scale case-control study is a drawback. However, this was a single-center study; therefore, in contrast to previous studies, we could maintain uniformity in treatment policies and operative and other procedural details. Considering the proportion of advanced CCC of all ovarian cancers, 28 patients in our study may not be small. (B) Potential histopathological misclassification cannot be excluded. However, CCC was diagnosed in the study population at our hospital by histopathologists familiar with gynecologic oncology. (C) Bev is contraindicated in patients with thromboembolic conditions. CCC is associated with thromboembolic diseases. However, we administered Bev without active thromboembolic events after adequate heparin prophylaxis against thromboembolic events. (D) We did not show the efficacy of Bev in overall survival. In the Bev− group, relapse occurred in 7 of 10 patients; 4 of these patients received Bev for relapse. Overall survival was not different between the groups in this retrospective study due to crossover to Bev. This result was similar to those of GOG 218 [9], which was a prospective study.

## 5. Conclusions

Since the introduction of Bev as a therapeutic option in patients with ovarian cancers, many studies have reported the use of biomarkers to evaluate the efficacy of Bev; however, no valid biomarkers are available in real-world practice. Advanced ovarian CCC is rare; therefore, it is difficult to determine the efficacy of Bev for this cancer. However, our study shows that CCC itself can serve as a biomarker for Bev. In this study, treatment for advanced CCC, as well as other histopathological types, included aggressive surgery to achieve complete resection without residual disease followed by paclitaxel and carboplatin chemotherapy concomitant with Bev administration to target the VEGF pathway. This approach might be an effective therapeutic strategy that prolonged PFS. We hope that our findings will be proven in adequate clinical trials. It would be interesting to find biomarkers to select the patients who may benefit from the treatment, in future studies. 

## Figures and Tables

**Table 1:** Patient Characteristics.

Characteristic	Bev− Group (*n* = 10)		Bev+ Group (*n* = 18)		*p* Value
Age					
Median age, years (IQR)	54 (48–59)		53 (46–63)		0.81
FIGO Stage, No. (%)					
III	7	70%	14	78%	0.674
IV	3	30%	4	22%	
Performance status, No. (%)					
0–1	9	90%	13	72%	0.375
2–4	1	10%	5	28%	
CA 125, IU/mL, (median, IQR)	419 (247–924)		271 (121–607)		0.179
Peritoneal Cancer Index (median, IQR)	5.5 (3–11.25)		5.5 (3–17.25)		0.727
Timing of debulking surgery					
Primary	10	100%	11	61%	
Interval	0	0%	6	33%	0.075
No debulking surgery	0	0%	1	6%	
Surgical complexity score, No. (%)					
Median, IQR	9 (4.8–11)		9 (4–14)		0.7
Low (0–3)	1	10%	2	11%	
Moderate (4–7)	3	30%	7	39%	0.874
High (8–18)	6	60%	9	50%	
Completeness of resection, No. (%)					
0 cm	9	90%	15	83%	
>0 cm	1	10%	2	11%	0.742
>1 cm	0		1	6%	
Firstline chemotherapy regimen					
Tri-weekly paclitaxel (or docetaxel) carboplatin	4	40%	0	0%	0.01
Weekly paclitaxel carboplatin	6	60%	18	100%	
Completeness of firstline chemotherapy, No. (%)					
Completeness	10	100%	17	94%	1
No-completeness	0		1	6%	

Abbreviations: FIGO, International Federation of Gynecology and Obstetrics; IQR, interquartile range: NACT, neoadjuvant chemotherapy.

**Table 2:** Adverse events induced by bevacizumab in bevacizumab+ group (*n =* 18).

Adverse Events	Grade									
	0		1		2		3		4	
Hypertension	4	22%	4	22%	6	33%	4	22%	0	
Proteinuria	8	44%	5	28%	1	6%	4	22%	0	
Bleeding	12	67%	5 ^a^	28%	0		1 ^b^	6%	0	
Colonic obstruction	16	89%	0	0%	0		2	11%	0	
Thromboemboic events	18		0		0		0		0	
Gastrointestinal perforation	18		0		0		0		0	
Wound dehiscence	18		0		0		0		0	
Fistula	18		0		0		0		0	

^a^: nasal bleeding, ^b^: anal hemorrhage.

**Table 3:** Multivariable Cox proportional analysis of risk factors for progression-free survival and overall survival for advanced clear cell carcinoma.

Variable		PFS			OS		
		Hazard Ratio	95% CI	*p* Value	Hazard Ratio	95% CI	*p* Value
Performance Status	0–1/≥2	0.28	0.09–0.94	0.049	0.33	0.10–1.03	0.058
Bevacizumab use	Bev +/−	0.26	0.08–0.82	0.022	0.52	0.18–1.46	0.216
Completeness of resection	Complete/Others	0.20	0.06–0.70	0.013	0.19	0.05–0.78	0.021

## Data Availability

All datasets analyzed during the current study are available from the corresponding author upon reasonable request.
